# Two-Step Synthesis of Bismuth-Based Hybrid Halide Perovskite Thin-Films

**DOI:** 10.3390/ma14247827

**Published:** 2021-12-17

**Authors:** Vanira Trifiletti, Sally Luong, Giorgio Tseberlidis, Stefania Riva, Eugenio S. S. Galindez, William P. Gillin, Simona Binetti, Oliver Fenwick

**Affiliations:** 1 School of Engineering and Materials Science (SEMS), Queen Mary University of London, Mile End Road, London E1 4NS, UK; s.luong@qmul.ac.uk (S.L.); e.s.suenagalindez@qmul.ac.uk (E.S.S.G.); o.fenwick@qmul.ac.uk (O.F.); 2 Department of Materials Science and Solar Energy Research Center (MIB-SOLAR), University of Milano-Bicocca, Via Cozzi 55, I-20125 Milan, Italy; giorgio.tseberlidis@unimib.it (G.T.); s.riva60@campus.unimib.it (S.R.); simona.binetti@unimib.it (S.B.); 3 Department of Physics and Astronomy, Queen Mary University of London, Mile End Road, London E1 4NS, UK; w.gillin@qmul.ac.uk

**Keywords:** bismuth, halide perovskites, thin-film, two-step, solution processing

## Abstract

Lead halide perovskites have been revolutionary in the last decade in many optoelectronic sectors. Their bismuth-based counterparts have been considered a good alternative thanks to their composition of earth-abundant elements, good chemical stability, and low toxicity. Moreover, their electronic structure is in a quasi-zero-dimensional (0D) configuration, and they have recently been explored for use beyond optoelectronics. A significant limitation in applying thin-film technology is represented by the difficulty of synthesizing compact layers with easily scalable methods. Here, the engineering of a two-step synthesis in an air of methylammonium bismuth iodide compact thin films is reported. The critical steps of the process have been highlighted so that the procedure can be adapted to different substrates and application areas.

## 1. Introduction

Bismuth-based hybrid halide perovskites are composed of elements widely available from natural sources and show improved chemical stability and less toxicity compared to their lead-based counterparts [1,2,3,4,5]. For these reasons, bismuth-based perovskites have emerged as a new research field, being explored for use in solar cells; however, the results obtained have not led to improvements in the state-of-the-art, due to the poor substrate coverage and to the charge confinement that limit their use in photovoltaics [3,6]. The stoichiometry, indeed, leads the structure to settle in a quasi-zero dimensional (0D) configuration, where the metal halide octahedra are almost isolated [3,7,8]. Their 0D electronic structure is highly explored for use in photodiodes [9], thermoelectric generators [10], and detectors [11]. Still, the production of a compact and continuous film remains challenging, and the best results are obtained by vapour-assisted deposition, even though the use of cheap and straightforward synthetic methods is a requirement becoming more and more crucial in the device design [3,12]. Unfortunately, the solution-processed thin films that have been reported so far are neither compact nor uniform [3,13,14,15,16]. Encouraging results on (CH_3_NH_3_)_3_Bi_2_I_9_ (MABI) deposition were obtained using two-step depositions on mesoporous TiO_2_ [17], where the BiI_3_ layer is dipped in a CH_3_NH_3_I (MAI) solution to complete the synthesis. However, the growth substrate has a decisive influence on the morphology of the final film: morphologies with vastly different features could be produced when varying the substrate from TiO_2_ (compact or mesoporous) to NiO, Al_2_O_3_ or PEDOT:PSS [3,18,19]. Here, a quartz-coated glass with high wettability and low roughness was used to reduce the substrate impact on the synthesis as much as possible. So far, BiI_3_ has been deposited by spin-coating [20], epitaxial electrodeposition [21], and thermal evaporation [22]. In this work, in order to produce a compact layer, BiI_3_ was deposited by thermal evaporation of commercial powders, while MAI was introduced by dipping in a solution in isopropyl alcohol (IPA). Chen et al. followed the same synthetic route [23], employing a spin-coated BiI_3_ thin film, but the resulting substrate coverage was incomplete [23]. This work defines a procedure developed and performed in air with optimised dipping and annealing times, leading to thick and compact films on flat substrates.

## 2. Materials and Methods

### 2.1. Materials

Bismuth(III) iodide (99%) and isopropyl alcohol (≥99.5%) were purchased from Sigma-Aldrich. Methylammonium iodide (98%) and ultra-flat quartz coated glass (2 × 1.5 cm) were purchased from Ossia Ltd. All salts and solvents were used as received without any further purification.

### 2.2. Synthesis of MABI Thin Films

The quartz-coated glasses were cleaned in an ultrasonic bath with IPA for 10 min. BiI_3_ was deposited by thermal evaporation at 10^−6^ mbar, with a deposition rate of 2 Å s^−1^ (crucible temperature of 170 °C). The thin films thus obtained were immersed in a 100 mL beaker containing 20 mL of the MAI solution in IPA (10 mg mL^−1^). Immersion times have been optimized during the work. Finally, the samples were annealed at 100 °C in a closed petri dish, set on a hot plate under a fume hood. Annealing times have been optimized during the work.

### 2.3. Characterization

Thin-film thickness was evaluated using a Bruker DektakXT profilometer, the standard deviation calculated on the average of 7 measurements at different points. The optical band gaps were evaluated by measuring the transmission spectra with a PerkinElmer Lambda 950 spectrometer equipped with an integrating sphere. The absorption coefficient (α) was calculated using the relation α(λ) = 1/*t* ln(1/T(λ)), where *t* is the sample thickness and T(λ) is the transmittance. The optical band gap was determined by plotting (αhν)^2^ versus hν and fitting the linear region of the absorption edge (OriginPro 2020b). X-ray diffraction (XRD) measurements were performed on a D5000 X-ray Powder Diffractometer (Siemens), using Cu-K_α_ radiation. Raman spectroscopy was performed using a Renishaw inVia confocal Raman microscope, equipped with a 785 nm laser; data analysis was performed by using OriginPro 2020b. X-ray photoelectron spectroscopy (XPS) measurements were performed with a Thermo Scientific™ Nexsa™ Surface Analysis System, and the XPS spectra were recorded and processed using the Thermo Avantage software. An FEI Inspect-F scanning electron microscope (SEM) was used for imaging at an accelerating voltage of 10 kV.

## 3. Results and Discussion

**Figure 1:**
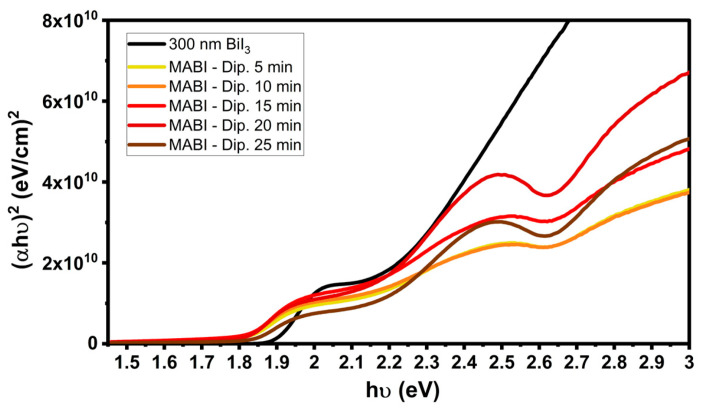
Tauc plots of the 300 nm BiI_3_ layer, and MABI films obtained after 5, 10, 15, 20, and 25 min of dipping in the MAI solution in IPA.

Different thicknesses of BiI_3_ and different dipping times were tested to obtain compact MABI films. The MAI solution in IPA was fixed at 10 mg mL^−1^ [23], and the BiI_3_ dipping was performed at room temperature. Annealing was performed on a hot plate at 100 °C for 90 min in air. The initial BiI_3_ film thickness and the BiI_3_ dipping time were varied, as summarized in Table 1. The main challenge of the method was the difficulty in obtaining a uniform transformation of the precursor layer. The homogeneity of the final MABI layer was evaluated by averaging the thicknesses measured with the profilometer. The standard deviation of the thickness was decisive in selecting the optimum growth conditions: with 300 nm—BiI_3_ films, thick layers were obtained with minimal deviations from the average. Figure 1 shows the Tauc plots of the produced films. The optical band gap was calculated from the linear fitting of the absorption edge. The extrapolated values agree with those reported in the literature: 1.90 eV for BiI_3_ film [24] and 1.80–1.82 eV for MABI [15]. The characteristic excitonic peak at 2.4 eV [15,24] becomes more or less evident according to the dipping time. A short immersion time (5 and 10 min) does not seem enough for complete conversion to the MABI structure; meanwhile, for immersion times longer than 15 min, the peak is well defined. Then, the subsequent characterisation was carried out to choose between 20 and 25 min.

**Figure 2:**
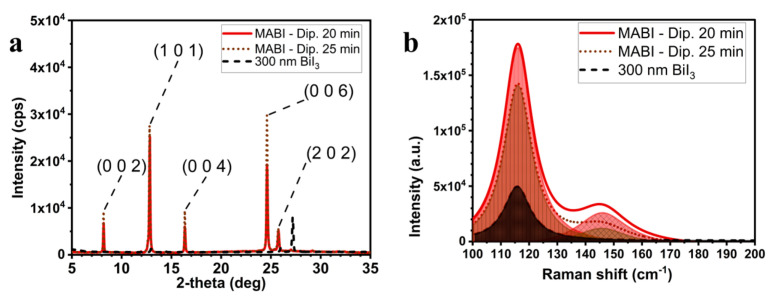
XRD patterns (**a**) and Raman spectra (**b**) of the 300 nm BiI_3_ layer, and MABI films obtained after 20 and 25 min dipping in the MAI solution in IPA.

The structures of the films dipped for 20 and 25 min belong to the hexagonal system with space group P6_3_/*mmc* (XRD patterns in Figure 2a) [25,26]. Raman analysis can be used to probe changes in Bi-I bond stretching when MAI coordinates it. Figure 2b shows in dark brown the single stretching mode visible at 116 cm^−1^ for pristine BiI_3_. When it is coordinated in the MABI structure, in addition to the peak at 116 cm^−1^, the stretching mode at 146 cm^−1^ emerges [27]. Signals at 115–117 cm^−1^ and 146 cm^−1^ have been assigned to the (Bi-I) stretching mode, and the second peak appears when CH_3_NH_3_I is coordinated to the metal centre [3,27]. The area ratio of 116 cm^−1^ to 146 cm^−1^ components (76/24 for 20 min of dipping; 84/16 for 25 min) suggests that the maximum number of coordinated metal centres is reached for 20 min immersion. On this basis, 20 min was taken to be the optimal immersion time.

**Figure 3:**
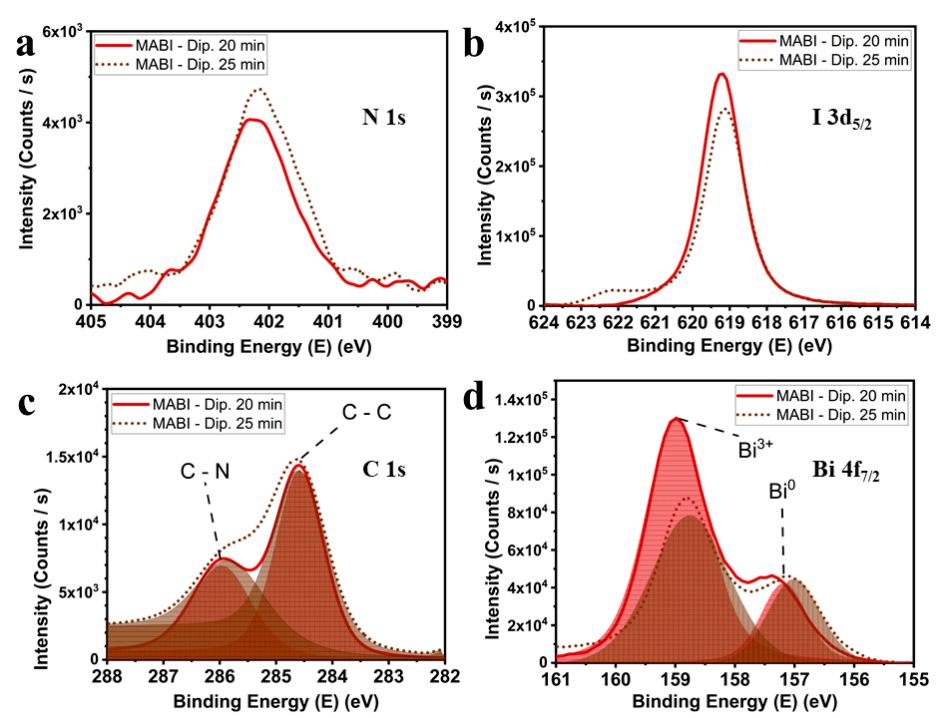
XPS high-resolution core-level spectra, related to the MABI films obtained after 20 and 25 min dipping in the MAI solution in IPA, for (**a**) N 1s; (**b**) I 3d_5/2_; (**c**) C 1s; (**d**) Bi 4f_7/2_.

The oxidation states of the chemical species were investigated by XPS, and the high-resolution core-level spectra for N, I, C, and Bi are presented in Figure 3. The N 1s spectra are composed of a broad peak around 402 eV (Figure 3a), confirming the presence of free amine group NH_2_, bonded to C, and the N-C bond [28]. The peak around 619 eV belongs to I 3d_5/2_ (Figure 3b). In the sample dipped for 25 min, a shoulder appearing at higher energy can be related to iodine oxidation at the surface [28]. The C 1s spectrum (Figure 3c) has two components, one given by the C-C bond at 284.6 eV and the other by the C-N in MABI at 286.0 eV [28]. XPS Bi 4f_7/2_ core-level shows that metallic bismuth (peaks at 157.0 eV) is present (Figure 3d). The reduction of metallic bismuth has been reported to be a consequence of X-ray irradiation [29,30]. The most likely mechanism for this is analogous to reported Pb^0^ evolution from MAPbI_3_ under the XPS environment, that is X-ray induced photolysis of any metal halide salts present in the film (PbI_2_ in the reported case, or BiI_3_ in our case). The emergence of Bi^0^ can therefore be linked to unreacted BiI_3_ in the films. Therefore, the annealing time was varied to improve the MABI stability in a challenging environment.

**Figure 4:**
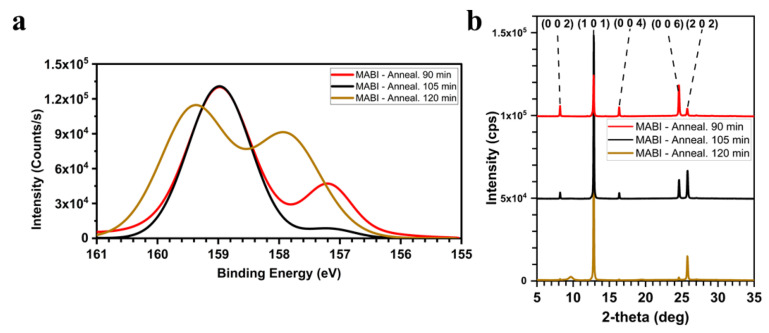
(**a**) XPS high-resolution core-level spectra for Bi 4f_7/2_, recorded for the MABI films obtained after 20 dipping in the MAI solution in IPA, and annealed at 100 °C for 90, 105, 120 min in air; (**b**) XRD patterns of the MABI films obtained after 20 dipping in the MAI solution in IPA, and annealed at 100 °C for 90, 105, 120 min in air.

**Figure 5:**
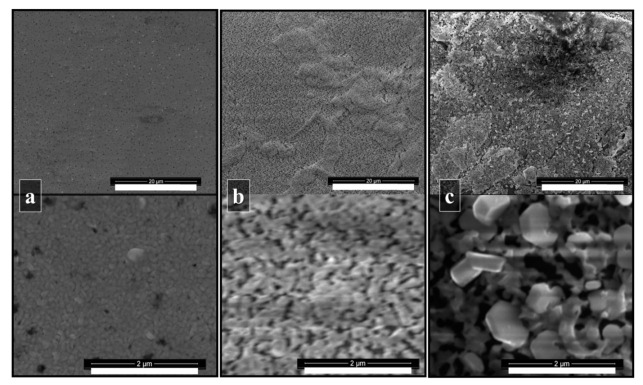
SEM images of (**a**) 300 nm BiI_3_ layer; (**b**) MABI film obtained with immersion time in CH_3_NH_3_I solution of 20 min and annealed at 100 °C for 90 min in air; (**c**) MABI film obtained with immersion time in CH_3_NH_3_I solution of 20 min and annealed at 100 °C for 105 min in air. Scale: 20 μm top and 2 μm bottom images.

Figure 4a shows the core-level spectra of Bi 4f_7/2_ after annealing at 100 °C for 90, 105, and 120 min: the sample treated for 105 min shows shallow Bi^0^ content. The shorter annealing time (90 min) probably leaves unreacted BiI_3_ in the film, which can be reduced to Bi^0^ under the X-ray beam. McGettrick et al. proved that in CH_3_NH_3_PbI_3_, the X-rays employed during the XPS measurement can trigger the photolysis of Pb^2+^ to Pb^0^, through light-induced generation of PbI_2_ [31]. The longer annealing time (120 min) results in a prominent Bi^0^ peak, which is likely due to MAI loss driving the formation of BiI_3,_ which can then be reduced to Bi^0^ under the X-ray beam. In addition, the XRD pattern of the 120 min annealed sample, Figure 4b, shows that the peaks at 8.2°, 16.3°, 24.6° have reduced to almost background levels. Meanwhile, a broad peak appears at 9.7°, suggesting the growth of an amorphous phase. Comparing the XRD patterns of the annealed sample for 90 and 105 min (Figure 4b), the (1 0 1) and (2 0 2) peaks’ intensities in the spectrum of the sample annealed for 105 min are more significant, and, as the layer thickness is similar (875 ± 75 nm for 105 min; 850 ± 60 nm for 90 min), an enhanced crystallinity is possible and a more preferential alignment. This is confirmed by the SEM images (Figure 5): hexagonal crystals, which were not present in the sample annealed for 90 min, are evident in the film annealed for 105 min.

## 4. Conclusions

A two-step synthesis in air of methylammonium bismuth iodide thin-films is reported, using a method based on the conversion of a thermally evaporated bismuth iodide layer by immersion into an MAI solution. The critical process steps, namely the dipping time in a methylammonium iodide solution and the final annealing, were controlled to obtain a compact thin-film of about 1 µm thickness. The fine-tuning of the dipping and annealing times can allow the synthesis to be moved from an inert glove box environment to a fume hood. The described perovskite layer is suitable for use in thin-film technologies; therefore, the reported results may enable the use of bismuth-based perovskites in the next technological device generation, in which quantum phenomena are exploited.

## Figures and Tables

**Table 1:** BiI_3_ film thickness, dipping times in MAI in IPA solution, and the obtained MABI film thicknesses.

BiI_3_—Thickness (nm)	Deviation (%)	Dipping Time (min)	MABI—Thickness (nm)	Deviation (%)
50	2	5	100	8
50	2	10	140	8
50	2	15	180	9
50	2	20	250	9
50	2	25	275	9
100	2	5	150	10
100	2	10	200	10
100	2	15	260	10
100	2	20	275	10
100	2	25	350	10
250	3	5	500	9
250	3	10	550	9
250	3	15	700	10
250	3	20	800	10
250	3	25	850	10
300	3	5	600	8
300	3	10	700	7
300	3	15	800	7
300	3	20	850	7
300	3	25	900	8

## Data Availability

The data presented in this study are available on request from the corresponding author.
